# Comparing perspectives on research needs from stakeholders vs. researchers in an exposome project

**DOI:** 10.1093/eurpub/ckac131.461

**Published:** 2022-10-25

**Authors:** S Benz, M Weber, P van den Hazel, S Jansen, A Arat, M White, N Riedel, G Ristovska, I van Kamp, D Schreckenberg

**Affiliations:** 1 ZEUS GmbH, Hagen, Germany; 2 City of Utrecht, Utrecht, Netherlands; 3 INCHES Network, Ellecom, Netherlands; 4 RIVM, Bilthoven, Netherlands; 5 Karolinska Institutet, Stockholm, Sweden; 6 Department of Social Epidemiology, Bremen University, Bremen, Germany; 7 Institute of Public Health of Republic of North Macedonia, Skopje, Republic of North Macedonia

## Abstract

**Background:**

Exposome research looks into how combined exposures affect human health. The EU-funded Equal-Life project focuses on physical and social exposures in a child’s environment and its effects on children’s mental health and cognitive development in the life course. Perspectives and priorities on what to study in particular might differ between practitioners and researchers. Therefore, collaboration with external stakeholders from various fields is encouraged to integrate practical experience and link it to the researchers’ aims.

**Methods:**

Two Delphi studies were conducted collecting and evaluating research questions to be studied in the project among a) the researchers within the project and b) among external stakeholders from various European countries. The exercise was to evaluate the research questions for group a) based on relevance and testability and for b) e.g. regarding practitioners’ work and options for policies. Involved stakeholders work in health care, and urban planning, among others. Prioritised questions are collated.

**Findings:**

Within the researchers’ group, top-rated questions were mainly mechanism-directed in terms of how and to what extent certain factors affect children’s mental health and cognitive development, cumulative effects in different settings, among others. Stakeholders most value research questions on practical issues, e.g. effects of early experiences of discrimination, critical windows in children’s lives that are most sensitive regarding the impact of exposures on mental health and cognitive development, or impact of exposures in early stages of life.

**Discussion:**

In comparing approaches of stakeholders and researchers, stakeholders’ input from the practical field can shape the approach of the research process. The second benefit is to derive implications for creating effective interventions and policies to prevent adverse effects of environmental exposures and to foster positive health in children and later on in life.

**Key messages:**

• Bi-directional exchange between researchers and external stakeholders can make gaps visible.

• Engaging stakeholders into a research process can help sharpening the aim and outcome of a project.

